# Supplementary material for: Empirical methods for controlling false positives and estimating confidence in ChIP-Seq peaks
Source: BMC Bioinformatics. 2008 Dec 5;9:523. doi: 10.1186/1471-2105-9-523 (PMC2628906; doi:10.1186/1471-2105-9-523)
Supplement: Additional File 1 — A variety of html documents from the USeq web site detailing the available applications, their best usage, output file type descriptions, command line menus, etc. [file 1471-2105-9-523-S1.zip › USeqUserGuides/JavaDoc.zip!/JavaDoc/util/bio/seq/package-summary.html]

util.bio.seq


|  |  |  |
| --- | --- | --- |
| **Overview** \| **Package** \| Class \| **Use** \| **Tree** \| **Deprecated** \| **Index** \| **Help** | |  |
| **PREV PACKAGE**   **NEXT PACKAGE** | **FRAMES**    **NO FRAMES**     **All Classes** |  |


---

## Package util.bio.seq

Nucleic acid sequence manipulation.

**See:**
  
          **Description**

| **Class Summary** | |
| --- | --- |
| **BinaryBooleanReaderWriter** | Can read or write a compressed boolean[] by converting each 8 booleans to a unique string and assigns them to byte. |
| **ConvertFasta2GCBoolean** | Converts fasta file(s) into serialized boolean[]s where every base g or c is true all others false. |
| **ConvertFasta2RealSeqBoolean** | Converts fasta file(s) into compressed binary boolean[]s where GATC is true all (NXgatcnx) others false. |
| **FetchIndexedSequence** | Fetches sequence given a directory of indexed sequences, see IndexFastas. |
| **IndexedSequence** |  |
| **IndexFastas** |  |
| **IndexRegions** |  |
| **LowerCase2Xs** | Converts a directory of files that contain lowercase masked sequences to Xs. |
| **MakeSpliceJunctionFasta** | Generates a fasta file for each. |
| **MakeTranscriptome** | Generates a fasta file for each gene model containing exon seqs plus splice junctions. |
| **MaskExonsInFastaFiles** | Takes a UCSC gene table and a directory of xxx.fasta files and replaces the exonic sequence with Xs. |
| **MergeFastas** | Merges many fasta's into one fasta with a defined number of X spacers. |
| **OligoTiler** | Tiles oligos across a list of genomic regions. |
| **ParseSeqsFromUCSCGeneTable** |  |
| **Seq** | For manipulating nucleic acid sequences. |

## Package util.bio.seq Description

Nucleic acid sequence manipulation.

---


|  |  |  |
| --- | --- | --- |
| **Overview** \| **Package** \| Class \| **Use** \| **Tree** \| **Deprecated** \| **Index** \| **Help** | |  |
| **PREV PACKAGE**   **NEXT PACKAGE** | **FRAMES**    **NO FRAMES**     **All Classes** |  |


---
